# Cognition in Virtual Reality: Assessing User Acceptability and Feasibility of Virtual Reality Cognitive Screening for Older Adults

**DOI:** 10.1002/alz70858_099841

**Published:** 2025-12-24

**Authors:** Frank Ho‐yin Lai, Benjamin K Yee, Eileen Han‐Jie Wang, Joe Butler, Andrew Graham, Eddie Yip‐kuen Hai, Cath Darling, Stephanie Whittington, Julie‐anne Lowe

**Affiliations:** ^1^ The Northumbria University Newcastle, UK, United Kingdom; ^2^ Mental Health Research Centre, The Hong Kong Polytechnic University, Hong Kong, Hong Kong; ^3^ The Hong Kong Polytechnic University, Hong Kong, Hong Kong; ^4^ The Hong Kong Polytechnic University, Hong Kong, China; ^5^ School of Psychology, University of Sunderland, Sunderland, United Kingdom; ^6^ School of Health & Life Sciences, Teesside University, Middlesbrough, United Kingdom; ^7^ Faculty of Health Sciences and Wellbeing, The Sunderland University, Sunderland, United Kingdom; ^8^ Northumbria Univeristy, Newcastle, Newcastle upon Tyne, United Kingdom; ^9^ Department of Social Work, Education and Community Wellbeing, Faculty of Health and Life Sciences, The Northumbria University, Newcastle, United Kingdom; ^10^ Northumbria University, Newcastle, United Kingdom

## Abstract

**Background:**

Cognitive health significantly influences the ageing journey, and virtual reality gamified tools offer innovative approaches for evaluating cognitive function.

**Method:**

This study explores the acceptability and feasibility of a Virtual Reality Working Memory Test (VRWMT), a gamified cognitive assessment tool in assessing spatial working memory (SWM), among three age groups—12 young adults (YA, 18–50 years old), 12 younger elderly (YE, 60–69 years old), and 12 older elderly (OE, 70+ years old) —using the Technology Acceptance Model (TAM) with constructs including perceived usefulness (PU), perceived ease of use (PEU), attitude toward using (ATU), and behavioural intention to use (BIU) to analyse users’ perspectives.

**Result:**

Findings reveal distinct perceptions shaped by age, technology familiarity, and socio‐demographic factors. YA participants value the tool for its productivity and cognitive enhancement capabilities, reporting high PEU and a positive ATU driven by engaging features. YE participants associate PU with cognitive health maintenance, emphasising the need for user‐friendly navigation and motivational feedback to support ATU. OE participants link PU to practical daily applications, with PEU heavily reliant on accessibility features such as large fonts and voice assistance. Their ATU improves with encouragement from caregivers and clear assurances of the tool's relevance. BIU across all groups depend on the tool's ability to integrate seamlessly into daily life and deliver measurable benefits.

**Conclusion:**

These findings underpin the necessity for a tailored approach in designing the VRWMT to meet the unique needs of each demographic. By incorporating adaptive interfaces, robust onboarding, and continuous user feedback, the VRWMT can become an inclusive and effective tool for cognitive health assessment and intervention, fostering long‐term user engagement and improved outcomes.

